# Complete genome sequence of *Terriglobus saanensis* type strain SP1PR4^T^, an *Acidobacteria* from tundra soil

**DOI:** 10.4056/sigs.3036810

**Published:** 2012-09-26

**Authors:** Suman R. Rawat, Minna K. Männistö, Valentin Starovoytov, Lynne Goodwin, Matt Nolan, Lauren Hauser, Miriam Land, Karen Walston Davenport, Tanja Woyke, Max M. Häggblom

**Affiliations:** 1 Department of Biochemistry and Microbiology, Rutgers, The State University of New Jersey, New Brunswick, New Jersey, 08901-8520, USA; 2 Finnish Forest Research Institute, Rovaniemi, Finland; 3 Department of Cell Biology and Neuroscience, Rutgers, The State University of New Jersey, Piscataway, New Jersey, USA.; 4 Oak Ridge National Laboratory, Oak Ridge, Tennessee, USA; 5 Los Alamos National Laboratory, Bioscience Division, Los Alamos, New Mexico, USA; 6 DOE Joint Genome Institute, Walnut Creek, California, USA

**Keywords:** cold adapted, acidophile, tundra soil, Acidobacteria

## Abstract

*Terriglobus saanensis* SP1PR4^T^ is a novel species of the genus *Terriglobus*. *T. saanensis* is of ecological interest because it is a representative of the phylum *Acidobacteria*, which are dominant members of bacterial soil microbiota in Arctic ecosystems. *T. saanensis* is a cold-adapted acidophile and a versatile heterotroph utilizing a suite of simple sugars and complex polysaccharides. The genome contained an abundance of genes assigned to metabolism and transport of carbohydrates including gene modules encoding for carbohydrate-active enzyme (CAZyme) family involved in breakdown, utilization and biosynthesis of diverse structural and storage polysaccharides. *T. saanensis* SP1PR4^T^ represents the first member of genus *Terriglobus* with a completed genome sequence, consisting of a single replicon of 5,095,226 base pairs (bp), 54 RNA genes and 4,279 protein-coding genes. We infer that the physiology and metabolic potential of *T. saanensis* is adapted to allow for resilience to the nutrient-deficient conditions and fluctuating temperatures of Arctic tundra soils.

## Introduction

Strain SP1PR4^T^ (= DSM 23119 = ATCC BAA-1853) is the type strain of *Terriglobus saanensis*. It is second of two validly ascribed species of the genus *Terriglobus,* with *T. roseus* first isolated from agricultural soils in 2007 [1]. *T. saanensis* SP1PR4^T^ was isolated from Arctic tundra soil collected from a wind exposed site of Saana fjeld, north-western Finland (69°01’N, 20°50’E) [2,3]. The species name *saanensis* (sa.a.nen' sis. N.L. masc. adj. saanensis) pertains to Mount Saana in Finland.

*Acidobacteria* are found in diverse soil environments and are widely distributed in Arctic and boreal soils [4-8]. However, relatively little is still known about their metabolic potential and ecological roles in these habitats. Despite a large collection of *Acidobacteria* 16S rRNA gene sequences in databases that represent diverse phylotypes from various habitats, few have been cultivated and described. *Acidobacteria* represent 26 phylogenetic subdivisions based on 16S rRNA gene phylogeny [9] of which subdivisions 1, 3, 4 and 6 are most commonly detected in soil environments [10]. The abundance of *Acidobacteria* has been found to correlate with soil pH [2,10,11] and carbon [1,12,13] with subdivision 1 *Acidobacteria* being most abundant in slightly acidic soils. The phylogenetic diversity, ubiquity and abundance of this group suggest that they play important ecological roles in soils.

Our previous studies on bacterial community profiling from Arctic alpine tundra soils of northern Finland have shown that *Acidobacteria* dominate in the acidic tundra heaths [2] and after multiple freeze-thaw cycles [6]. Using selective isolation techniques, including freezing soils at -20°C for 7 days, we have been able to isolate several slow growing and fastidious strains of *Acidobacteria*. On the basis of phylogenetic, phenotypic and chemotaxonomic data, including 16S rRNA, rpoB gene sequence similarity and DNA–DNA hybridization, strain SP1PR4^T^ was classified as a novel species of the genus *Terriglobus* [3]. Here, we summarize the physiological features together with the complete genome sequence and annotation of *Terriglobus saanensis* SP1PR4^T^.

## Classification and features

**Figure 1:**
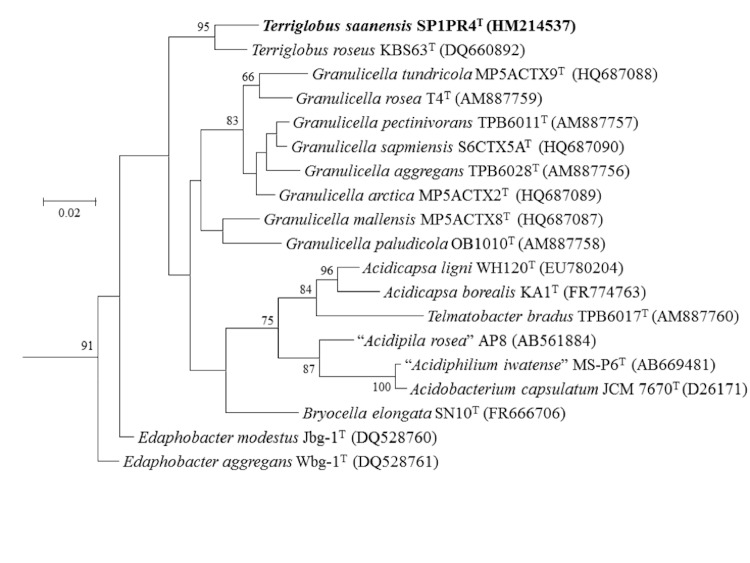
Phylogenetic tree highlighting the position of *T. saanensis* SP1PR4^T^ relative to the other type strains within the family *Acidobacteriaceae*. The maximum likelihood tree was inferred from 1,359 aligned positions of the 16S rRNA gene sequences and derived using MEGA version 5 [14]. Bootstrap values (expressed as percentages of 1,000 replicates) of >50 are shown at branch points. Bar: 0.02 substitutions per nucleotide position. The strains (type strain=T) and their corresponding GenBank accession numbers are displayed in parentheses with strain *T. saanensis* SP1PR4^T^ shown in bold. *Bryobacter aggregatus* MPL3 (AM162405) was used as outgroup. *T. saanensis* SP1PR4^T^ and *T. roseus* KBS 63^T^ (DSM 18391) genome sequences have been revealed.

Within the genus *Terriglobus*, two species are ascribed with validly published names, *T. saanensis* SP1PR4^T^ [3] isolated from Arctic tundra soils and *T. roseus* KBS 63^T^ (DSM 18391) isolated from agricultural soils (KBS-LTER site) [1]. Searching the NCBI non-redundant nucleotide database for homology to 16S rRNA gene sequence of *T. saanensis* SP1PR4^T^ identified 10 cultured and 20 uncultured strains that were unclassified, with ≥97% 16S rRNA sequence identity. Phylogenetic tree based on 16S rRNA gene depicting the position of *T. saanensis* SP1PR4^T^ relative to the other type strains within the family *Acidobacteriaceae* is shown in Figure 1. *T. saanensis* SP1PR4^T^ is distinctly clustered into a separate branch with *T. roseus* KBS 63^T^ (DQ660892) [1], as its closest described relative (97.1% 16S rRNA sequence identity). Strain SP1PR4^T^ showed ~95% 16S rRNA gene identity to four strains in the genus *Granulicella* isolated from tundra soils, namely “*G. tundricola”* (95.9%), “*G. sapmiensis” (*95.8%), “*G. mallensis”* (95.5%) and “*G. arctica*” (94.9%) [3,15] (Figure 1).

**Figure 2:**
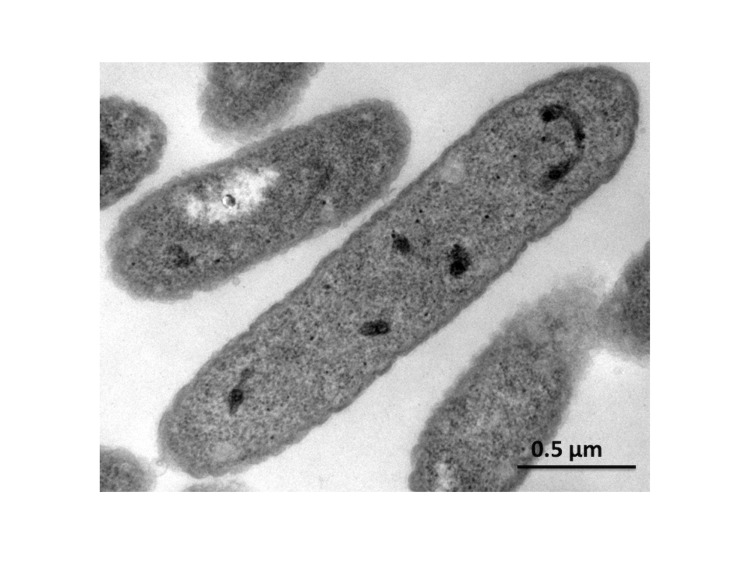
Electron micrograph of cells of *T. saanensis* strain SP1PR4^T^ (bar 0.5 µm).

Strain SP1PR4^T^ grows at pH 4.5-7.5 with an optimum at 6.0 and at temperatures of +4 to +30°C with an optimum of +25°C on R2 medium [3]. On R2 agar, strain SP1PR4^T^ forms small, circular, convex colonies with a diameter of approximately 1 mm. The pigment varies from light beige to light pink depending on the age of the culture. Cells of strains SP1PR4^T^ are Gram-negative, non-spore-forming, non-motile aerobic rods with a length of 1.5– 3.0 µm and a diameter of 0.5–0.7 µm. The cell-wall structure in ultrathin sections of electron micrographs of cells of strain SP1PR4^T^ demonstrates numerous outer-membrane vesicles (Table 1, Figure 2).

**Table 1:** Classification and general features of *T. saanensis* SP1PR4^T^ according to the MIGS recommendations [16].

**MIGS ID**	**Property**	**Term**	**Evidence codes**
	Classification	Domain *Bacteria*	TAS [17]
		Phylum *Acidobacteria*	TAS [18,19]
		Class *Acidobacteria*	TAS [20]
		Order *Acidobacteriales*	TAS [21,22]
		Family *Acidobacteriaceae*	TAS [18,23]
		Genus *Terriglobus*	TAS [1]
		Species *Terriglobus saanensis*	TAS [3]
		Type strain: SP1PR4^T^	
	Gram stain	negative	TAS [3]
	Cell shape	rod	TAS [3]
	Motility	non-motile	TAS [3]
	Sporulation	non-spore forming	TAS [3]
	Temperature range	4–30°C	TAS [3]
	Optimum temperature	25°C	TAS [3]
	pH range	4.5-7.5	TAS [3]
	Optimum pH	6.0	TAS [3]
	Salinity	not reported	NAS
MIGS-22	Oxygen requirement	aerobe	TAS [3]
	Carbon source	cellobiose, D-fructose, D-galactose, D-glucose, lactose, D-maltose, D-mannose, D-ribose, sucrose, D-trehalose, D-xylose, D-melezitose, D-raffinose, starch, pectin, laminarin and aesculin	TAS [3]
MIGS-6	Habitat	terrestrial	TAS [3]
MIGS-15	Biotic relationship	free-living	TAS [3]
MIGS-14	Pathogenicity	non-pathogen	NAS
	Biosafety level	1	NAS
	Isolation	tundra soil	TAS [3]
MIGS-4	Geographic location	Saana fjeld, Arctic tundra, Finland	TAS [3]
MIGS-5	Sample collection time	2004-2005	TAS [3]
MIGS-4.1	Latitude	69°01’N,	TAS [3]
MIGS-4.2	Longitude	20°50’E	TAS [3]
MIGS-4.3	Depth	not reported	NAS
MIGS-4.4	Altitude	not reported	NAS

*Evidence codes - IDA: Inferred from Direct Assay (first time in publication); TAS: Traceable Author Statement (i.e., a direct report exists in the literature); NAS: Non-traceable Author Statement (i.e., not directly observed for the living, isolated sample, but based on a generally accepted property for the species, or anecdotal evidence). These evidence codes are from the Gene Ontology project [24].

Strain SP1PR4^T^ utilized carbon substrates for growth which include cellobiose, D-fructose, D-galactose, D-glucose, lactose, D-maltose, D-mannose, D-ribose, sucrose, D-trehalose, D-xylose, D-melezitose, D-raffinose and N-acetyl-D-glucosamine. Strain SP1PR4^T^ hydrolyzed polysaccharides such as starch, pectin, laminarin and aesculin but not gelatin, cellulose, xylan, lichenan, sodium alginate, pullulan, chitosan or chitin. Enzyme activities of strain SP1PR4^T^ include chitobiase, catalase, acid and alkaline phosphatase, leucine arylamidase, naphthol-AS-B1-phosphohydrolase, α- and β-galactosidase, α- and β-glucosidase, β-glucuronidase, N-acetyl-β-glucosaminidase, α-mannosidase and α-fucosidase [3,15].

### Chemotaxonomy

The major cellular fatty acids in *T. saanensis* SP1PR4^T^ are iso-C_15:0_ (39.9%), C_16:1 ω7c_ (28.4%), iso-C_13:0_ (9.8%) and C_16:0_ (9.8%). The cellular fatty acid compositions of strain SP1PR4^T^ were relatively similar to that of *T. roseus* DSM 18391^T^, with higher relative abundance of iso-C_13:0_ and a corresponding lower abundance of iso-C_15:0_ in strain SP1PR4^T^ [3].

## Genome sequencing and annotation

### Genome project history

Strain SP1PR4^T^ was selected for sequencing in 2009 by the DOE Joint Genome Institute (JGI) community sequencing program. The Quality Draft (QD) assembly and annotation were completed on August 6, 2010. The complete genome was made available on Jan 24, 2011. The genome project is deposited in the Genomes On-Line Database (GOLD) [25] and the complete genome sequence of strain SP1PR4^T^ is deposited in GenBank. Table 2 presents the project information and its association with MIGS version 2.0 [16].

**Table 2:** Genome sequencing project information.

**MIGS ID**	**Property**	**Term**
MIGS 31	Finishing quality	Finished
MIGS-28	Libraries used	Three libraries, an Illumina GAii shotgun library (GSGY), a 454 Titanium standard library (GSXT, GWTA) and a paired end 454 (GSFP) library
MIGS 29	Sequencing platforms	454 Titanium standard, 454 Paired End, Illumina
MIGS 31.2	Sequencing coverage	39× (454), 180× (Illumina)
MIGS 30	Assemblers	Newbler, Velvet, Phrap
MIGS 32	Gene calling method	ProdigaL, GenePRIMP
	Locud Tag	AciPR4
	INSDC / RefSeq ID	CP002467, NC_014963,
	GenBank Date of Release	October 7, 2011
	GOLD ID	Gc01604
	NCBI project ID	48971
MIGS 13	Source material identifier	ATCC BAA-1853, DSM 23119
	Project relevance	Environmental, Biogeochemical cycling of carbon, Biotechnological, GEBA

### Growth conditions and genomic DNA extraction

Strain SP1PR4^T^ was cultivated in R2 medium as previously described [3]. Genomic DNA (gDNA) of high sequencing quality was isolated using a modified CTAB method and evaluated according to the Quality Control (QC) guidelines provided by the DOE Joint Genome Institute.

### Genome sequencing and assembly

The finished genome of *T. saanensis* SP1PR4^T^ (JGI ID 4088690) was generated at the DOE Joint genome Institute (JGI) using a combination of Illumina [26] and 454 technologies [27]. For this genome, an Illumina GAii shotgun library which generated 23,685,130 reads totaling 916 Mb, a 454 Titanium standard library which generated 409,633 reads and a paired end 454 library with an average insert size of 10.8 kb which generated 180,451 reads totaling 157 Mb of 454 data, were constructed and sequenced. All general aspects of library construction and sequencing performed at the JGI can be found at the JGI website [28]. The 454 Titanium standard data and the 454 paired end data were assembled together with Newbler, version 2.3. Illumina sequencing data was assembled with Velvet, version 0.7.63 [29]. We integrated the 454 Newbler consensus shreds, the Illumina Velvet consensus shreds and the read pairs in the 454 paired end library using parallel phrap, version SPS - 4.24 (High Performance Software, LLC). The software Consed [30,31] was used in the finishing process. Illumina data was used to correct potential base errors and increase consensus quality using the software Polisher developed at JGI (Alla Lapidus, unpublished). Possible mis-assemblies were corrected using gapResolution (Cliff Han, unpublished), Dupfinisher [32], or sequencing cloned bridging PCR fragments with sub-cloning. Gaps between contigs were closed by editing in Consed, by PCR and by Bubble PCR (J-F Cheng, unpublished) primer walks. The final assembly is based on 157 Mb of 454 data which provides an average 39× coverage and 916 Mb of Illumina data which provides an average 180× coverage of the genome.

### Genome annotation

Genes were identified using Prodigal [33] as part of the Oak Ridge National Laboratory genome annotation pipeline, followed by a round of manual curation using the JGI GenePRIMP pipeline [34]. The predicted CDSs were translated and used to search the National Center for Biotechnology Information (NCBI) non-redundant database, UniProt, TIGRFam, Pfam, PRIAM, KEGG, (COGs) [35,36], and InterPro. These data sources were combined to assert a product description for each predicted protein. Non-coding genes and miscellaneous features were predicted using tRNAscan-SE [37], RNAMMer [38], Rfam [39], TMHMM [40], and signalP [41]. Additional gene prediction analysis and functional annotation were performed within the Integrated Microbial Genomes Expert Review (IMG-ER) platform [42].

## Genome properties

**Figure 3:**
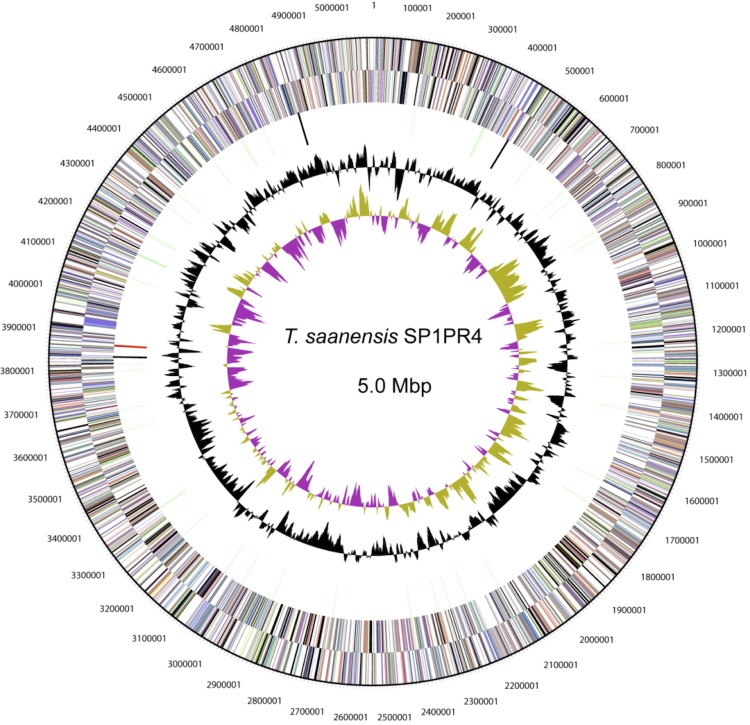
Graphical representation of circular map of the chromosome of *T. saanensis* strain SP1PR4^T^ displaying relevant genome features. From outside to center: Genes on forward strand (color by COG categories), genes on reverse strand (color by COG categories), RNA genes (tRNAs green, rRNAs red, other RNAs black), GC content, GC skew.

The genome consists of one circular chromosome of 5,095,226 bp in size with a GC content of 57.3% and consists of 54 RNA genes (Figure 3, Table 3). Of the 4,333 predicted genes, 4,279 are protein-coding genes (CDSs) and 99 are pseudogenes. Of the total CDSs, 67% represent COG functional categories and 43% consist of signal peptides. The distribution of genes into COG functional categories is presented in Figure 3 and Table 4.

**Table 3:** Genome statistics

**Attribute**	**Value**	**% of Total**
Genome size (bp)	5,095,226	100%
DNA coding (bp)	4,578,206	89.9%
DNA G+C (bp)	2,921,371	57.3%
Number of replicons	1	100%
Total genes	4,334	100%
RNA genes	54	1.3%
rRNA operons	1	-
Protein coding genes	4,180	98.8%
Pseudo genes	99	2.3%
Genes with function prediction	3,203	73.9%
Genes in paralog clusters	2,220	51.2%
Genes assigned to COGs	3,170	73.2%
Genes with Pfam domains	3,108	71.7%
Genes with signal peptides	1,867	43.1%
Genes with transmembrane helices	1,082	25%
CRISPR repeats	0	-

**Table 4:** Number of genes associated with general COG functional categories.

**Code**	**Value**	**%age**	**Description**
J	163.0	4.6	Translation, ribosomal structure and biogenesis
A	2.0	0.1	RNA processing and modificatin
K	293.0	8.3	Transcription
L	142.0	4.0	Replication, recombination and repair
B	0.0	0.0	Chromatin structure and dynamics
D	24.0	0.7	Cell cycle control, Cell division, chromosome partitioning
Y	0.0	0.0	Nuclear structure
V	98.0	2.8	Defense mechanisms
T	174.0	4.9	Signal transduction mechanisms
M	307.0	8.7	Cell wall/membrane biogenesis
N	56.0	1.6	Cell motility
Z	2.0	0.1	Cytoskeleton
W	0.0	0.0	Extracellular structures
U	113.0	3.2	Intracellular trafficking and secretion
O	122.0	3.4	Posttranslational modification, protein turnover, chaperones
C	196.0	5.5	Energy production and conversion
G	303.0	8.6	Carbohydrate transport and metabolism
E	243.0	6.9	Amino acid transport and metabolism
F	69.0	2.0	Nucleotide transport and metabolism
H	134.0	3.8	Coenzyme transport and metabolism
I	116.0	3.3	Lipid transport and metabolism
P	134.0	3.8	Inorganic ion transport and metabolism
Q	85.0	2.4	Secondary metabolites biosynthesis, transport and catabolism
R	443.0	12.5	General function prediction only
S	323.0	9.1	Function unknown
-	1163.0	26.8	Not in COGs

## Discussion

Genome analysis of *T. saanensis* identified a high abundance of genes assigned to COG functional categories for transport and metabolism carbohydrates (9.5%) and amino acids (7.6%), energy conversion (6.2%), cell envelope biogenesis (9.6%) and transcription (9.2%) [15]. This indicates that the *T. saanensis* genome encodes for functions involved in transport and utilization of nutrients, mainly carbohydrates and amino acids for energy production and cell biogenesis to maintain cell integrity in cold tundra soils. Further genome analysis revealed an abundance of gene modules for glycoside hydrolases, glycosyl transferases, polysaccharide lyases, carbohydrate esterases, and non-catalytic carbohydrate-binding modules within the carbohydrate-active enzymes (CAZy [43]) family involved in breakdown, utilization and biosynthesis of carbohydrates [15]. *T. saanensis* hydrolyzed complex carbon polymers, including pectin, laminarin, and starch, and utilized sugars such as cellobiose, D-mannose, D-xylose, D-trehalose and laminarin. This parallels genome predictions for CDSs encoding for enzymes such as pectinases, chitinases, alginate lyases, trehalase and amylases. *T. saanensis* was unable to hydrolyze carboxymethyl cellulose (CMC) on plate assays and lacked CDSs encoding for cellulases involved in cellulose hydrolysis. However, the *T. saanensis* genome contained a BcsZ gene encoding for an endocellulase (GH8) as part of a bacterial cellulose synthesis (bcs) operon involved in cellulose biosynthesis in several species. This operon consists of clusters of genes in close proximity to the BcsZ gene which includes a cellulose synthase gene (*bcsAB),* a cellulose synthase operon protein (*bcsC)* and a cellulose synthase operon protein (*yhj)* [15]. In addition, the *T. saanensis* genome encoded for a large number of gene modules representing glycosyl transferases (GTs) involved in carbohydrate biosynthesis which include cellulose synthase (UDP-forming), α-trehalose phosphate synthase [UDP-forming], starch glucosyl transferase, ceramide β-glucosyltransferase involved in biosynthesis of cellulose, trehalose, starch, hopanoid, and capsular/free exopolysaccharide (EPS) [15]. This suggests that *T. saanensis* is involved in hydrolysis of lignocellulosic soil organic matter, utilization of stored carbohydrates and biosynthesis of exopolysaccharides. Therefore, we surmise that *T. saanensis* may be central to carbon cycling processes in Arctic and boreal soil ecosystems.
